# Drying Behavior and Kinetics of Drying Process of Plant-Based Enteric Hard Capsules

**DOI:** 10.3390/pharmaceutics13030335

**Published:** 2021-03-05

**Authors:** Chuqi He, Haodong Wang, Yucheng Yang, Yayan Huang, Xueqin Zhang, Moses Arowo, Jing Ye, Na Zhang, Meitian Xiao

**Affiliations:** 1 College of Chemical Engineering, Huaqiao University, Xiamen 361021, China; he-sylvi@stu.hqu.edu.cn (C.H.); ishdwang@126.com (H.W.); yangyc@hqu.edu.cn (Y.Y.); yyhuang@hqu.edu.cn (Y.H.); xqzhang2009@hqu.edu.cn (X.Z.); yejenny@hqu.edu.cn (J.Y.); 2 Xiamen Engineering and Technological Research Center for Comprehensive Utilization of Marine Biological Resources, Xiamen 361021, China; 3 Department of Chemical & Process Engineering, Moi University, 3900-30100 Eldoret, Kenya; musarowo@yahoo.com

**Keywords:** plant-based enteric hard capsules, hot-air drying, drying characteristics, modeling

## Abstract

The drying process is a significant step in the manufacturing process of enteric hard capsules, which affects the physical and chemical properties of the capsules. Thus, the drying characteristics of plant-based enteric hard capsules were investigated at a constant air velocity of 2 m/s in a bench scale hot-air dryer under a temperature range of 25 to 45 °C and relative humidity of 40 to 80%. Results indicate that the drying process of the capsules mainly occur in a falling-rate period, implying that moisture transfer in the capsules is governed by internal moisture diffusion rate. High temperature and low relative humidity reduce drying time but increase the drying rate of the capsules. Investigation results of the mechanical properties and storage stability of the capsules, however, reveal that a fast drying rate leads to plant-based enteric hard capsules of low quality. Scanning electron microscopy further demonstrates that more layered cracks appear in capsules produced under a faster drying rate. The Page model yielded the best fit for describing thin-layer drying of the capsules based on the coefficient of determination and reduced chi-square. Moreover, it was established that the effective moisture diffusivity of the capsules increases with an increase in drying temperature or reduction in relative humidity.

## 1. Introduction

The word capsule originates from the Latin word “capsula” and its original meaning is “small box.” The development of capsules has nearly 173 years of history since James Murdock patented the two-piece hard capsules in 1847 [1]. Enteric hard capsules have attracted attention because of their role in targeted drug release [2], where they are designed to remain intact in the stomach and then release the active substance in the upper intestine [3]. Nonetheless, gelatin capsules have deficient physicochemical properties and safety issues such as risks of animal disease [4], cross-linking with aldehyde materials and ability to get soft and brittle under extreme conditions [5,6,7]. Moreover, the process of coating enteric material on the gelatin capsule’s surface is complicated so that the distribution of enteric coated materials on the surface of gelatin capsules is poor [8]. Meanwhile enteric coated materials are also relatively expensive [9]. Therefore, much attention has been paid to the development of plant-based enteric hard capsules [10]. The enteric coated materials that have been developed for plant-based capsules are usually anionic polymethacrylate, cellulose acetate phthalate or polyvinyl acetate phthalate. Huyghebaert et al. had used each of the three materials to make enteric-coated hydroxypropyl methylcellulose (HPMC) capsules [11]. But calcified coating is one of the emerging simple methods of preparing plant-based enteric hard capsules [12] with stable enteric solubility [13]. To the best of our knowledge, information that plant-based enteric hard capsules was prepared by calcified coating is hitherto rare.

The manufacturing process of plant-based enteric hard capsules is similar to that of other hard capsules and mainly involves dipping model pins, drying, shell drawing, cutting, nesting, and so forth. All of these operations, except drying, are mainly controlled by mechanical processing capability. Therefore, the drying process is a significant step as it induces changes in the structure of the capsule material, and thus affects the physical and chemical properties of the enteric hard capsules [14,15]. Up to now, there is limited information on the drying process of plant-based enteric hard capsules. Consequently, a systematic study on drying behavior and the kinetics of the drying process of plant-based enteric hard capsule is necessary.

Industrial drying techniques include hot-air drying, microwave drying, infrared drying, vacuum freeze drying, and so forth. Hot-air drying is the most common technique for industrial and commercial drying of food and pharmaceutical engineering industry [16] owing to its mature equipment, low cost, simplicity and strong adaptability [17,18,19]. However, improper drying conditions may lead to massive defects such as warping, blisters and cracks [20,21] and consequently products of low quality. Ashikin et al. [22] established that the alginate film plasticity can be modulated by varying drying temperature, and noted that the film plasticity can greatly be improved at a drying temperature of 80 °C due to air bubble formation and reduced alginate molecular weight in the film. Liu et al. [23] systematically studied the moisture transfer of hot-air drying of soft gelatin capsules and established that decreasing the air relative humidity and a gradual increase in drying temperature are proper drying conditions. In contrast, improper drying conditions resulted in adhesion and crack of soft gelatin capsules.

Apart from experimental observation, it is necessary to establish a mathematical model to predict and describe the kinetics of the drying process [24]. Thin-layer drying models can be categorized as theoretical, semi-theoretical and empirical models. Lewis model [25], also known as exponential model, is a theoretical model based on Newton’s law of cooling and describes the movement of water molecules. The model neglects the movement of internal water molecules and mainly considers the resistance of boundary layer on material surface to the movement of moisture diffusion. The Page model [26], Henderson and Pabis model [27], and Logarithmic model [28] are all semi-empirical models based on diffusion theory. According to the second Fick’s law, assuming that moisture in the material diffuses outward from the surface in the form of liquid moisture, these models are obtained by partial modification of the theoretical model. On the other hand, the Wang and Singh model [29] is an empirical model based on experimental data. The model does not take into account the heat and moisture transfer during the drying process and its parameters have no practical physical significance. However, to the present day, these models are still usually used in the description of moisture transfer in materials [30].

Overall, considering the importance of the drying process to plant-based enteric hard capsules and limited information on drying kinetics of plant-based capsules thin-layer drying process, the work herein aims to systematically analyze the influence of different drying conditions such as drying temperature and relative humidity on moisture transfer and quantity of plant-based enteric hard capsules. The tested capsules were composed of HPMC, sodium alginate, xanthan gum, gellan gum and potassium citrate. Moreover, five different conventional mathematical models have been employed to validate the experimental data. Finally, the effective moisture diffusivity and activation energy of the drying plant-based enteric hard capsules are obtained. The results will provide a theoretical basis and guidance for prediction of hot-air drying process control and optimization of alginate plant-based enteric hard capsules.

## 2. Materials and Methods

### 2.1. Materials

Commercially available food-grade HPMC (HT-E15), calcium chloride and potassium citrate were obtained from Shandong Ruitai Co., Ltd. (Shandong, China) while sodium alginate, xanthan gum and gellan gum were supplied by Shanghai Macklin Biochemical Co., Ltd. (Shanghai, China).

### 2.2. Methods

#### 2.2.1. Preparation of Plant-Based Enteric Hard Capsule

**Figure 1:**
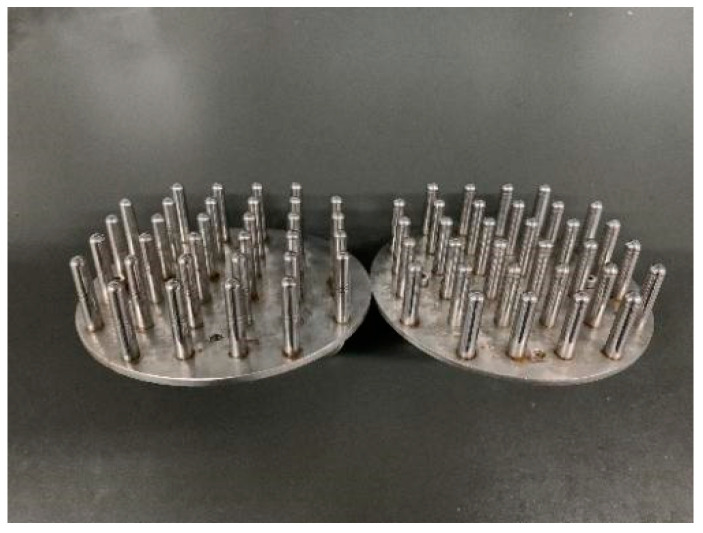
Stainless steel mold pins of capsules (cylindrical: cap, 6.8 ± 0.02 mm; body, 6.4 ± 0.02 mm).

A solution mixture was prepared by dissolving xanthan gum (0.4%, *w*/*w*), gellan gum (0.4%, *w*/*w*) and sodium alginate (0.7%, *w*/*w*) in distilled water containing potassium citrate (0.3%, *w*/*w*). The mixture was then heated to 80 °C under moderate agitation, which was at 480 r/min. HPMC, the film-forming agent (9%, *w*/*w*) was added to the mixed solution under agitation and then maintained at 80 °C for 40 min to ensure complete dissolution. Afterwards, the solution was degassed using a circulating water vacuum pump for 2 h and then cooled to 60 °C for 4 h to ensure stability. The capsules were prepared using the well-established method [12] of dipping stainless steel mold pins (Figure 1) into the homogeneous solution at 47 °C. The stainless steel mold pins which were adhered with capsule solutions were immersed immediately in calcium chloride solution for 20 s to get calcified after dipping in order to achieve enteric effect. Finally, the capsules were dried in a dryer at constant temperature and constant relative humidity.

#### 2.2.2. Drying Process

The stainless steel mold pins used in the drying process, which were adhered with capsules solutions, were uniformly placed on the shelf of the dryer. Hot air at a velocity of 2 m/s entered into the dryer and flowed upwards across the pins. The drying temperature and relative humidity were measured by temperature and humidity transducers, respectively.

As shown in Table 1, nine operating conditions of drying plant-based enteric hard capsules were performed at varying drying temperature: 25, 30, 35, 40 and 45 °C, and relative humidity: 40%, 50%, 60%, 70% and 80%. According to our previous research, it was found that at a drying temperature of 35 °C, without overheating but higher than the room temperature, the drying time is appropriate. At the same time, the relative humidity of 60% is widely used in industry. At each operating condition, samples of plant-based enteric hard capsules all covered with a piece of stainless steel mold pin were instantly weighed at an interval of 10 min during the period of drying until a stable weight was achieved. Moreover, samples were placed in the air dryer at a fixed temperature of 105 °C for 12 h in order to get the dry matter mass. Drying experiments were measured in triplicate at different drying temperature and relative humidity, and the average experimental errors were less than 2%.

#### 2.2.3. Quality Measurement

##### The Breakage Test

Samples of 50 capsules obtained from the same drying condition were put into a glass tube measuring 24 and 200 mm in diameter and length, respectively. Then, a disc made of poly tetra fluoroethylene with a diameter of 22 mm weighing 20.0 ± 0.1 g was freely dropped from the mouth of the glass tube. The capsules were then observed to determine any breakage. The process was performed in triplicate at each drying condition. According to the China Pharmacopoeia [31], the capsules are unqualified if more than five tested capsules are broken.

##### The Disintegration Time Test

Samples of six capsules were filled with talc powder and were then put into a hanging basket of the lifting disintegrating instrument without baffle for testing according to the China Pharmacopoeia [31]. Firstly, the capsules were put into a simulated gastric fluid (hydrochloric acid and pepsin, pH = 1.0) and tasted for 2 h. Each capsule should be checked for any crack, disintegration, or softening. The basket was then removed and washed with a small amount of distilled water. Each tube was fixed with baffles and examined in a simulated intestinal fluid (phosphate buffer, pH = 6.8). The capsules were checked for disintegration during one hour period.

##### The Storage Stability Test

The storage stability of the capsules was tested in a container at a temperature of 20 ± 2 °C and relative humidity 85 ± 2% during 64 h. Samples of five plant-based enteric hard capsules were respectively filled with the same amount of fructose weighing 0.140 ± 0.005 g, and then weighed for every 8 h. The results were calculated as follows [31]:

(1) $$\mathrm{Moisture}\;\mathrm{Absorption}\;\mathrm{Ratio}=\;\frac{m_{t}-m_{0}}{m_{0}},$$

where *m*_0_ and *m_t_* respectively represent the mass of plant-based enteric hard capsules with fructose at initial and *t* time. The tests were also performed in triplicate at each drying condition.

#### 2.2.4. SEM

The plant-based enteric hard capsules obtained at different drying conditions were frozen with liquid nitrogen, and then gently knapped. A portion of the samples was fixed vertically on the sample table for gold spraying, then scanned using a HITACHI S-4800 scanning electron microscope (SEM). The acceleration voltage during the test was 10 kV. Among them, the amplification times of different drying conditions was 10,000 times.

#### 2.2.5. Modeling of the Thin-Layer Drying Data

The kinetics for thin-layer drying of plant-based enteric hard capsules were evaluated in the form of moisture ratio (MR) according to Equation (2).

(2) $$MR=\frac{M-M_{e}}{M_{0}-M_{e}},$$

where *M* represents the moisture content of plant-based enteric hard capsules (g water/g dry matter) at drying time *t*, *M*_0_ is the initial moisture content of plant-based enteric hard capsules (g water/g dry matter) and *M_e_* is the equilibrium moisture content of plant-based enteric hard capsules (g water/g dry matter).

Drying rate (*DR*) was calculated according to Equation (3).

(3) $$DR=\frac{M_{t2}-M_{t1}}{t_{2}-t_{1}},$$

where *M_t_*_1_ and *M_t_*_2_ respectively represent moisture contents between two sequential times while *t_1_* and *t*_2_ represent the corresponding time [32].

Five thin-layer drying models illustrating the drying process as set by many scholars were selected to represent the drying characteristics of plant-based enteric hard capsules as shown in Table 2. Experimental drying data were fitted to the selected drying models using non-linear least square method in Origin software (OriginLab, version 2016). A suitable drying model was selected based on the coefficient of determination (*R*^2^) and the reduced chi-square (*χ*^2^) [33]. The parameters *R*^2^ and *χ*^2^ were calculated according to Equations (4) and (5) respectively.

(4) $$R^{2}=1-\frac{\sum_{i=1}^{N}{\left(MR_{pre,i}-MR_{exp,i}\right)}^{2}}{\sum_{i=1}^{N}{\left(MR_{pre,mean}-MR_{exp,i}\right)}^{2}}$$

(5) $$\chi^{2}=\frac{\sum_{i=1}^{N}{\left(MR_{exp,i}\;-\;MR_{pre,i}\right)}^{2}}{N-2},$$

where *MR_exp_*_,*i*_ represents the experimental moisture ratio found in measurement and *MR_pre_*_,*i*_ represents predicted moisture ratio for this measurement. *N* is the number of observations.

#### 2.2.6. Calculation of the Effective Moisture Diffusivity

The mechanisms of moisture transfer during drying can be modelled mathematically from the second Fick’s law [34] according to Equation (6).

(6) $$\frac{\partial MR}{\partial t}=D_{eff}\left(\frac{\partial^{2}MR}{\partial x^{2}}\right),$$

where *D_eff_* represents the effective moisture diffusivity (m^2^/s). If the initial moisture distribution of the material is uniform, the moisture diffusion coefficient is constant throughout the drying process, and the shrinkage of the material is not considered, the moisture diffusion was assumed as one dimensional mass transport in hot-air drying.

The boundary conditions are as follows—*MR* is one, when *t* and *x* are 0. When *x* is variable, there are two cases: when *t* is zero, *MR* is a function and when *t* is infinity, *MR* is 0.

For the case of thin-layer drying, the thickness *L* can be regarded as a constant. Thus, one of the solutions was proposed as follows [35]:

(7) $$MR=\frac{M_{t}-M_{e}}{M_{0}-M_{e}}=\frac{8}{\pi^{2}}\sum_{n=0}^{\infty }\frac{1}{{\left(2n+1\right)}^{2}}exp\left[-{\left(2n+1\right)}^{2}\frac{\pi^{2}D_{eff}}{L^{2}}t\right],$$

where *L* represents the thickness of plant-based enteric hard capsules, and has a value of 2 × 10^−6^ m. For long drying times and neglecting the higher order term by setting *n* = 1 [36], the equation can be simplified as follows:

(8) $$MR=\frac{8}{\pi^{2}}exp\left(-\frac{\pi^{2}D_{eff}}{L^{2}}t\right).$$

Effective moisture diffusivity can be calculated by taking natural logarithm of both sides of Equation (8).

(9) $$\ln\;MR=ln\frac{8}{\pi^{2}}-\frac{\pi^{2}D_{eff}}{L^{2}}t.$$

Thus, *D_eff_* was deduced by plotting experimental drying data in accordance with ln *MR* versus drying time according to Equation (9), and the plot produced a straight line with a slope as follows:

(10) $$\mathrm{Slop}=-\frac{\pi^{2}D_{eff}}{L^{2}}.$$

#### 2.2.7. Calculation of the Activation Energy

The activation energy indicates the amount of energy required by 1 mol of water molecules to evaporate during their transfer through the materials. The relationship of the effective moisture diffusivity with temperature can be described by the Arrhenius-type equation according to Equation (11) [37].

(11) $$D_{eff}=D_{0}exp\left(-\frac{E_{a}}{RT_{a}}\right),$$

where *D*_0_ represents the pre-exponential factor of the Arrhenius equation (m^2^/s), *E_a_* represents the activation energy (J/mol), *R* represents the universal gas constant (kJ/mol K), and *T_a_* represents the absolute temperature (K). Activation energy can be calculated by taking natural logarithm of both sides of Equation (11) as follows:

(12) $$\ln\;\left(D_{eff}\right)=\ln\;\left(D_{0}\right)-\frac{E_{a}}{RT_{a}}.$$

Thus, *E_a_* was deduced by plotting experimental drying data in accordance with ln (*D_eff_*) versus the reciprocal of the absolute temperature (1/*T_a_*) according to Equation (12), and the plot produced a straight line with a slope as follows:

(13) $$\mathrm{Slop}=-\frac{E_{a}}{R}.$$

## 3. Results and Discussion

### 3.1. Drying Characteristics of Plant-Based Enteric Hard Capsules

**Figure 2:**
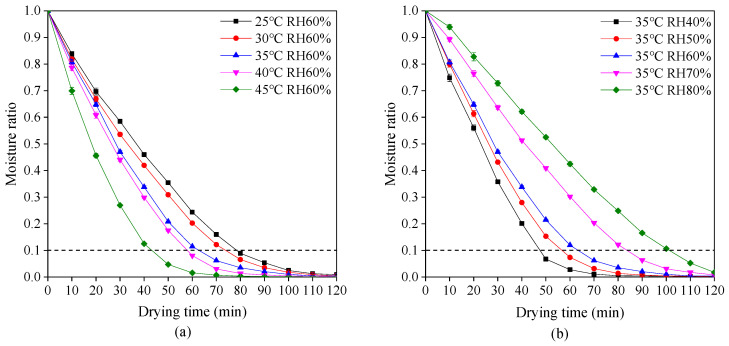
Drying behavior of plant-based enteric hard capsules at different (**a**) drying temperature and (**b**) relative humidity (RH).

The variations of moisture ratio with drying time at different drying temperatures and relative humidity are shown in Figure 2. The moisture ratio rapidly decreases during the early stage of drying and then decreases gently in the late stage. This phenomenon is in accordance with drying characteristics of most materials [38,39]. According to Chinese Pharmacopeia, the moisture ratio of dried plant-based hard capsules should not exceed 0.1. Hence, it is obvious in Figure 2 that an increase in drying temperature or reduction in relative humidity causes the required reduction of drying time. The drying time decreased from 79 to 43 min with an increase in drying temperature from 25 to 45 °C at a relative humidity of 60%. Due to the increase of drying temperature, the moisture on the surface of the capsules evaporates quickly while the moisture inside the capsules and the surface form a large gradient, making the moisture transfer faster. The drying time decreased from 103 to 48 min with a decrease in relative humidity from 60% to 40% at a drying temperature of 35 °C. Due to the reduction of relative humidity, the difference between the partial pressure of water vapor on the surface of the capsule and the ambient vapor pressure becomes large, which makes the moisture transfer faster and thus shortens the drying time.

**Figure 3:**
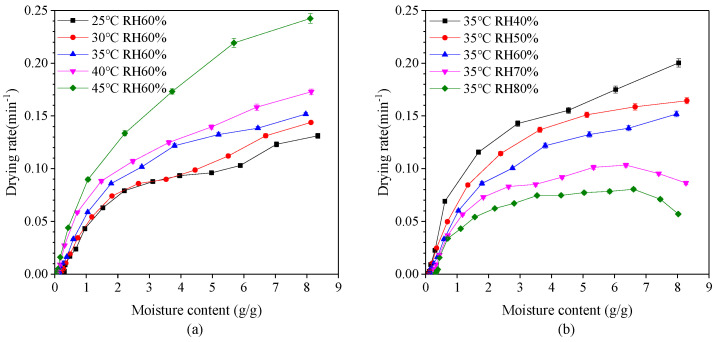
Effect of moisture content on the drying rate of plant-based enteric hard capsules at different (**a**) drying temperature and (**b**) relative humidity (RH).

The variation of drying rate with moisture content at different drying temperatures and relative humidity is shown in Figure 3. It is evident that the drying rate generally decreases with a reduction in moisture content. Drying rate also decreases with a decrease in drying temperature and an increase in relative humidity. The drying process mainly occurs in the falling-rate period which can be divided into two parts. When the moisture content of dry basis was between 2.0 g/g and 8.0 g/g, the first stage of falling-rate period appeared with the rate of reduction relatively slow. At this stage, the rate of vaporization from the surface of the capsule into the environment is greater than the rate of migration of water molecules from the interior of the capsule to the surface. At this drying stage, the wetted area on the surface of the capsule decreases and so the drying rate decreases. In other word, the first stage is mainly the surface drying of capsules. When the moisture content of dry basis was less than 2.0 g/g, the second stage falling-rate period appeared with the rate of reduction relatively fast. The reduction of drying rate is faster during the second stage falling-rate period than the first stage, since a section of moisture transfer moves to the inside of the capsule during the drying process, and the heat required for migration needs to be transferred to the transfer section through the dried capsules layer. Also, the transferred moisture enters the air through the capsules layer. The drying rate eventually decreases indicating that the internal moisture diffusion is the dominant factor. The second stage can be considered as the inside drying of capsules.

It is also evident that at a relative humidity greater than 70%, there exist two distinct drying periods—constant-rate period and falling-rate period, whereby the drying rate firstly tends to increase and then decrease. This phenomenon is in agreement with results of drying process of yam slices [40,41]. In the constant-rate period, the surface of the capsules is fully wetted. The rate of migration of water molecules from the interior to the surface is greater than the rate of vaporization from the surface into the environment, which is controlled by the rate of water vaporization on the surface.

### 3.2. Effect of Drying Conditions on the Quality of Plant-Based Enteric Hard Capsules

**Figure 4:**
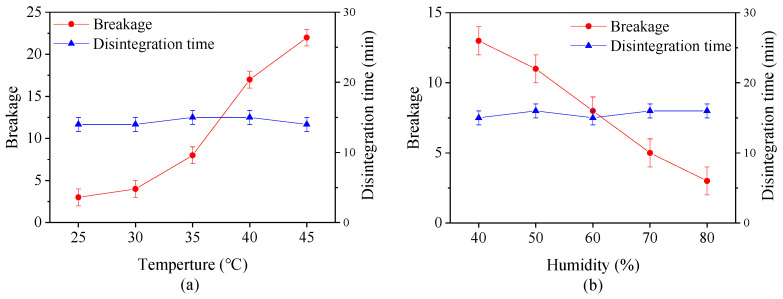
Effect of (**a**) drying temperature and (**b**) relative humidity on breakage and disintegration time of plant-based enteric hard capsules.

Figure 4 shows the effect of drying temperature and the relative humidity on the breakage and digestion time of capsules with same moisture content. It is evident that the breakage increases with an increase in drying temperature and a reduction in relative humidity. At temperature beyond 35 °C, more than 5 capsules were broken, indicating that the capsules under this condition are unqualified. This is probably because the moisture on the capsule surface rapidly evaporates at high drying temperature or low relatively humidity. At the same time, the moisture inside the capsules hardly transfer to the surface on time, making the bound water or immobilize water which binds with the material to disappear in these zones hence weak connections between the polysaccharide molecules [42,43]. All of these factors change the mechanical property of capsules. However, the digestion time of capsules is not significantly affected by different drying conditions, suggesting that the digestion property is closely related to the calcified coating process.

**Figure 5:**
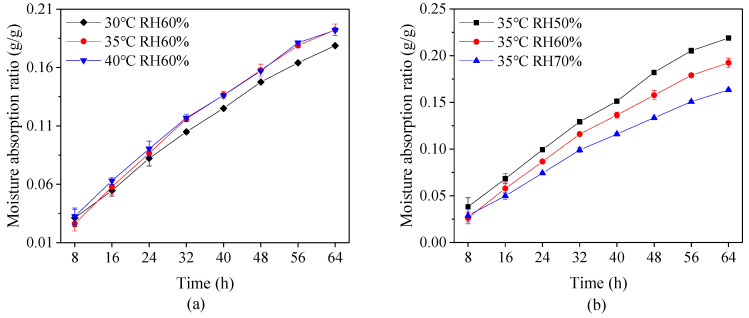
Moisture absorption ratio of capsules at different (**a**) drying temperature and (**b**) relative humidity (RH).

The capsules were filled with fructose, which is a moisture prone material, and then reserved in the environment at 20 ± 2 °C and relative humidity 85 ± 2% in order to measure the storage stability of the capsules. Figure 5 shows that the variation of moisture absorption ratio of the capsules under different drying conditions during 64 h period. It is evident that a longer duration leads to higher moisture absorption ratio, and the ratio at a high drying temperature or low relative humidity is higher than that at the low drying temperature or high relative humidity. This phenomenon suggests that improper drying conditions causes water to easily transfer through the capsule shell. This is probably because the rapid loss of moisture in capsules during a high drying temperature or low relative humidity leads to weak connection between layers and hence the loose form of material network structure.

**Figure 6:**
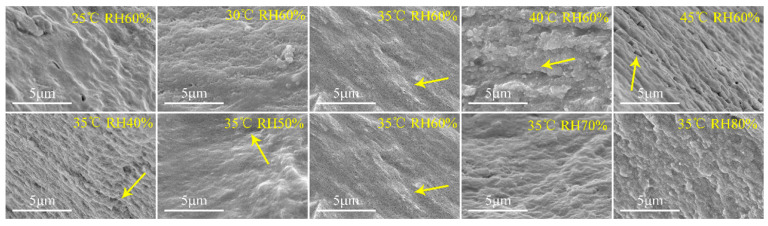
Scanning electron microscopy (SEM) images of plant-based enteric hard capsule sections at different (**top**) drying temperatures and (**bottom**) relative humidity.

The results of microscopic change in the plant-based enteric hard capsules at different drying temperatures and relative humidity as observed by SEM are shown Figure 6. At drying temperature between 25 to 30 °C, the capsules did not exhibit obvious cavities or cracks. However, the stratification phenomenon and cracks on the section of the capsules were obvious at drying temperature beyond 30 °C. Relative humidity also influences the microscopic nature of the capsules. At a relative humidity of 40%, cracks and layers appeared on capsule material section, and the phenomenon gradually disappeared with increase in relative humidity. The SEM images of the capsules further prove that the layered structure make the no-dense structure of the capsule shell, which subsequently decrease their mechanical and storage stability.

### 3.3. Formatting of Mathematical Components

Modelling the drying operation is important as it can help predict the moisture ratio of materials during the drying process [44]. The five different models applied to the drying curves of plant-based enteric hard capsules are listed in Table 2. All of the tested models reveal great values of *R*^2^, ranging from 0.9340 to 0.9994 (seen in Tables S1 and S2), and thus can adequately be used to describe the drying process. It is evident that the values of *R*^2^ of the Page model are all greater than 0.9940 under various drying temperature and relative humidity while those of *χ*^2^ are all less than 6.9495 × 10^−4^. The Page model has the highest *R*^2^ and the lowest *χ*^2^, making it the best model that accurately describes the thin-layer drying process of plant-based enteric hard capsules.

The drying constants of k and n in the Page model were developed as functions of drying temperature (*T*) and relative humidity (*U*). Using the multiple linear regression method in SPSS software (IBM, version 23), the regression equation of parameters *k* and *n* excluding the non-significant influencing factors (*p* < 0.05) in the Page equation were calculated as follows:

(14) $$k=0.048-0.003T+0.016U+4.696\times 10^{-5}T^{2}-0.03U^{2}$$

(*R*^2^ = 0.921, *p* < 0.05)

(15) $$n=1.521+0.046T-3.432U+0.001T^{2}+3.293U^{2}$$

(*R*^2^ = 0.887, *p* < 0.05).

Therefore, the Page model of hot-air drying of plant-based enteric hard capsules can be described as follows:

(16) $$MR=\exp\left(-\left(0.048+0.016U-0.003T-0.03U^{2}+4.696\times 10^{-5}T^{2}\right)t^{1.521-3.432U+0.046T+3.293U^{2}+0.001T^{2}}\right).$$

### 3.4. Effective Moisture Diffusivity and Activation Energy

Table 3 and Table 4 present the effective moisture diffusivity of the capsules under different drying conditions. According to Equation (8), the effective moisture diffusivity of plant-based enteric hard capsules vary from 1.6244 × 10^−10^ to 2.6618 × 10^−10^ m^2^/s and 2.4102 × 10^−10^ to 1.1940 × 10^−10^ m^2^/s, respectively. The values of *D_eff_* increase with increase in drying temperature but decrease with increase in relative humidity. A high drying temperature increases the activity of water molecules and consequently leads to high effective moisture diffusivity [38]. A lower relative humidity causes larger moisture gradient, and thus, greater driving force of drying results into higher effective moisture diffusivity.

**Figure 7:**
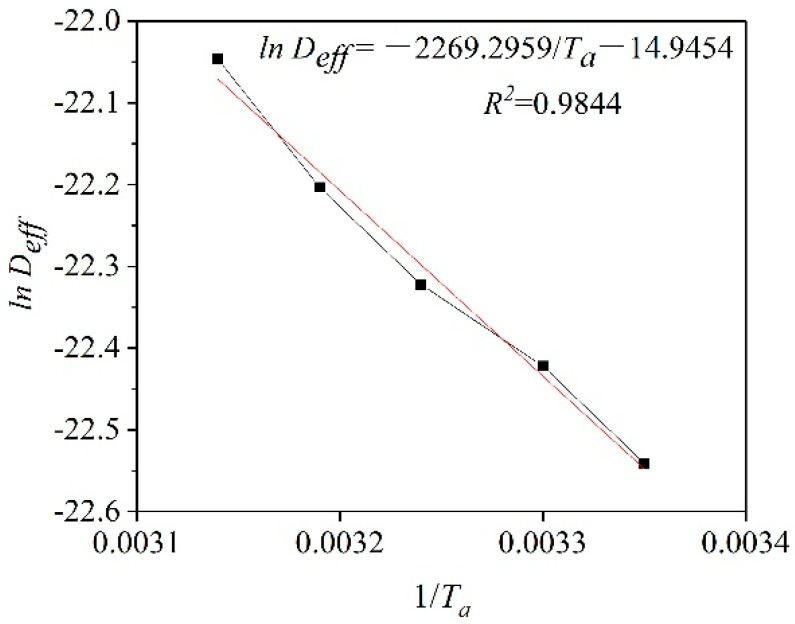
ln *(D_eff_*) versus the reciprocal of the absolute temperature (1/*T_a_*).

The activation energy for plant-based enteric hard capsules was obtained from the slope of the straight line in Figure 7 as 18.87 kJ/mol Equation (11). The value is lower compared to that of some materials [45,46] since capsules have a loose internal structure for easy transfer of moisture [47].

## 4. Conclusions

Drying is an important unit operation in the production of plant-based enteric hard capsules. The study herein investigated the drying characteristics of plant-based enteric hard capsules at a constant air velocity of 2 m/s in a bench scale hot-air dryer at a temperature range of 25 to 45 °C and relative humidity of 40 to 80%.

The investigations arrived at the following findings:

(1) Drying rate of the capsules increases with an increase in drying temperature and a reduction in relative humidity. The drying process mainly occur in falling-rate period, except under constant-rate period when the relative humidity is greater than 70%, implying that the moisture transfer in the capsules is governed by internal moisture diffusion rate.

(2) Increasing drying temperature or reducing the relative humidity decreases the quality of the capsules, leading to high chance of breakage of the capsules and reduction in their storage stability. However, drying condition has negligible influence on the digestion ability of the capsules. The SEM images of the capsules further prove that these conditions lead to layered structure and cracks in the capsules, a phenomenon which may be the main reason for reduction in the quality of the capsules. 

(3) Statistical analyses show that the Page model fits well with the experimental data. The effective moisture diffusivity of the capsules vary from 1.6244 × 10^−10^ to 2.6618 × 10^−10^ m^2^/s and 2.4102 × 10^−10^ to 1.1940 × 10^−10^ m^2^/s with an increase in drying temperature and relative humidity, respectively. The activation energy of the plant-based enteric hard capsules was established as 18.87 kJ/mol.

## Figures and Tables

**Table 1:** Experimental setup of different operating conditions.

Experimental Group	Fixed Factor	Experimental Factor	
The first group	relative humidity 60% air velocity 2 m/s.	drying temperature (°C)	25
			30
			35
			40
			45
The second group	drying temperature 35 °C air velocity 2 m/s.	relative humidity (%)	40
			50
			60
			70
			80

**Table 2:** Mathematical models applied to the thin-layer drying curves.

No.	Equation	Model	References
1	*MR = exp(−kt)*	Lewis	(K. Lewis, 1921)
2	*MR = exp(−kt^n^)*	Page	(Page, 1949)
3	*MR = a exp(−kt)*	Henderson and Pabis	(Henderson, 1961)
4	*MR = a exp(−kt) + c*	Logarithmic	(Toğrul, 2002)
5	*MR = 1 + at + bt* ^2^	Wang and Singh	(Wang, 1978)

**Table 3:** Effective moisture diffusivity (*D_eff_*) of the plant-based enteric hard capsules at different drying temperature.

Relative Humidity (%)	Drying Temperature (°C)	Effective Moisture Diffusivity (m^2^/s)
60	25	1.6244 × 10^−10^
60	30	1.8289 × 10^−10^
60	35	2.0212 × 10^−10^
60	40	2.2760 × 10^−10^
60	45	2.6618 × 10^−10^

**Table 4:** Effective moisture diffusivity (*D_eff_*) of the plant-based enteric hard capsules at different relative humidity.

Relative Humidity (%)	Drying Temperature (°C)	Effective Moisture Diffusivity (m^2^/s)
40	35	2.4102 × 10^−10^
50	35	2.2991 × 10^−10^
60	35	2.0212 × 10^−10^
70	35	1.6017 × 10^−10^
80	35	1.1940 × 10^−10^

## Data Availability

Not applicable.

## Supplementary Materials

The following are available online at https://www.mdpi.com/1999-4923/13/3/335/s1, Table S1: Results of statistical analysis of the mathematical models at different drying temperature, Table S2: Results of statistical analysis of the mathematical models at different relative humidity.
