# Supplementary material for: Drying Behavior and Kinetics of Drying Process of Plant-Based Enteric Hard Capsules
Source: Pharmaceutics. 2021 Mar 5;13(3):335. doi: 10.3390/pharmaceutics13030335 (PMC7998449; doi:10.3390/pharmaceutics13030335)
Supplement: Supplementary file 1 [file pharmaceutics-13-00335-s001.pdf]

pharmaceutics

MDPI

Supplementary Materials: Drying Behavior and Kinetics of Drying Process of Plant-Based Enteric Hard Capsules

Chuqi H, Haodong Wang, Yucheng Yang, Yayan Huang, Xueqin Zhang, Moses Arowo, Jing Ye, Na Zhang and Meitian Xiao

Table S1. Results of statistical analysis of the mathematical models at different drying temperature.

		Model														
**RH %**	T °C	Lewis			Page			Henderson and Pabis			Logarithmic			Wang and singh		
		parameters	R^2	χ^2	parameters	R^2	χ^2	parameters	R^2	χ^2	parameters	R^2	χ^2	parameters	R^2	χ^2
	25	k=0.0230	0.9688	0.0036	k=0.0050 n=1.3876	0.9940	6.9495×10^{-4}	a=1.0650 k=0.0244	0.9716	0.0033	a=1.2413 k=0.0159 c=-0.2199	0.9932	7.9250×10^{-4}	a=-0.0166 b=6.9156×10^{-5}	0.9985	1.6949×10^{-4}
	30	k=0.0253	0.9748	0.0029	k=0.0067 n=1.3428	0.9947	6.0882×10^{-4}	a=1.0572 k=0.0266	0.97674	0.0027	a=1.1782 k=0.0189 c=-0.1573	0.9935	7.4369×10^{-4}	a=-0.0181 b=8.2198×10^{-5}	0.9994	7.3465×10^{-5}
**60**	35	k=0.0295	0.9756	0.0028	k=0.0069 n=1.3908	0.9977	2.7376×10^{-4}	a=1.0637 k=0.0311	0.9781	0.0026	a=1.1324 k=0.0248 c=-0.0939	0.9887	0.0013	a=-0.0206 b=1.0498×10^{-5}	0.9968	3.7511×10^{-4}
	40	k=0.0322	0.9760	0.0028	k=0.0078 n=1.3884	0.9963	4.3090×10^{-4}	a=1.0585 k=0.0338	0.97768	0.0026	a=1.1162 k=0.0277 c=-0.0796	0.9874	0.0015	a=-0.0219 b=1.1636×10^{-5}	0.9953	5.4817×10^{-4}
	45	k=0.0453	0.9871	0.0014	k=0.0158 n=1.3157	0.9985	1.6062×10^{-4}	a=1.0380 k=0.0467	0.98747	0.0013	a=1.0601 k=0.0430 c=-0.0302	0.9902	0.0010	a=-0.0260 b=1.5474×10^{-4}	0.9452	0.0057

Table S2. Results of statistical analysis of the mathematical models at different relative humidity.

T °C	RH %	Model														
		Lewis			Page			Henderson and Pabis			Logarithmic			Wang and Singh		
		parameters	R^2	χ^2	parameters	R^2	χ^2	parameters	R^2	χ^2	parameters	R^2	χ^2	parameters	R^2	χ^2
	40	k=0.0383	0.9754	0.0028	k=0.0086 n=1.4289	0.9957	4.8913×10^{-4}	a=1.0545 k=0.0400	0.9764	0.0027	a=1.0921 k=0.0350 c=-0.0514	0.9820	0.0020	a=-0.0243 b=1.3889 $\times 10^{-4}$	0.9787	0.0024
	50	k=0.0327	0.9740	0.0031	k=0.0067 n=1.4377	0.9979	2.4231×10^{-4}	a=1.0662 k=0.0345	0.9764	0.0028	a=1.1213 k=0.0286 c=-0.0757	0.9855	0.0017	a=-0.0222 b=1.1895 $\times 10^{-4}$	0.9935	7.7057×10^{-4}
**35**	60	k=0.0295	0.9756	0.0028	k=0.0069 n=1.3908	0.9977	2.7376×10^{-4}	a=1.0637 k=0.0311	0.9781	0.0026	a=1.1324 k=0.0248 c=-0.0939	0.9887	0.0013	a=-0.0206 b=1.0498 $\times 10^{-4}$	0.9968	3.7511×10^{-4}
	70	k=0.0206	0.9512	0.0060	k=0.0023 n=1.5408	0.9958	5.1694×10^{-4}	a=1.0957 k=0.0224	0.9590	0.0050	a=1.4090 k=0.0122 c=-0.3684	0.9911	0.0011	a=-0.0146 b=5.0685 $\times 10^{-4}$	0.9942	7.0821×10^{-4}
	80	k=0.0159	0.9340	0.0077	k=0.0011 n=1.6372	0.9952	5.5207×10^{-4}	a=1.1108 k=0.0178	0.9474	0.0061	a=2.2280 k=0.0053 c=-1.1918	0.9959	4.7206×10^{-4}	a=-0.0105 b=1.6833 $\times 10^{-4}$	0.9948	5.9872×10^{-4}
